# The effects of repetitive transcranial magnetic stimulation in obese females with binge eating disorder: a protocol for a double-blinded, randomized, sham-controlled trial

**DOI:** 10.1186/s12888-015-0569-8

**Published:** 2015-08-12

**Authors:** Mara Fernandes Maranhão, Nara Mendes Estella, Maria Elisa Gisbert Cury, Veruska Lastoria Amigo, Clarissa Mollinero Picasso, Arthur Berberian, Iain Campbell, Ulrike Schmidt, Angélica Medeiros Claudino

**Affiliations:** 1 Eating Disorder Unit, Psychiatry Department, Universidade Federal de São Paulo (UNIFESP), R. Borges Lagoa, 570, 7th floor, CEP 04038–020, São Paulo, SP Brazil; 2 Laboratory Integrative Neuroscience (LiNC), Psychiatric Department, Universidade Federal de São Paulo (UNIFESP), R. Pedro de Toledo, 669, 3rd floor, CEP 04039–032, São Paulo, SP Brazil; 3 Section of Eating Disorder, Institute of Psychiatry, King’s College London, Denmark Hill, SE5 8AF, London, UK

**Keywords:** Binge eating disorder, Transcranial magnetic stimulation, Neuroimaging, Hormones, Eating disorders, Obesity, Control inhibition, Eating disorders

## Abstract

**Background:**

Binge eating disorder is a new category in DSM-5 and highly associated with higher body mass index. The neural mechanisms that underlie binge eating are of great interest in order to improve treatment interventions. Brain mechanisms underlying drug and food craving are suggested to be similar: for example, both are reported to be associated with increased neural activity in the orbitofrontal and anterior cingulate cortex, and a diminished regulatory influence from lateral prefrontal circuits. Several studies have begun to assess the potential benefits of brain stimulation in reducing craving and addictive behaviors. Data from a study of a one-off session of transcranial magnetic stimulation in healthy women identified as strong cravers and of individuals with bulimic-type eating disorders, reported a reduction in food craving and binge eating episodes. This provides support for a more extensive investigation of the potential therapeutic benefits of transcranial magnetic stimulation. Lastly, brain imaging studies and a dimensional approach, will improve understanding of the neural correlates of the disorders and of the mode of action of transcranial magnetic stimulation.

**Methods/Design:**

Sixty eligible obese females, with binge eating disorder, will be randomly allocated to receive 20 sessions of transcranial magnetic stimulation intervention (*n* = 30) or the sham transcranial magnetic stimulation intervention (*n* = 30) scattered 3 days/week. Thirty eligible controls will complete the baseline assessment. The primary outcome (number of binge eating episodes) will be assed at each treatment sessions, and 8 weeks after intervention completion (follow-up). It is hypothesized that mean weekly binge-eating episodes will be reduced in the intervention group, compared to the sham group, and that the effect will be maintained at follow-up.

**Discussion:**

Despite the severity associated with Binge Eating Disorder, there are limited treatment options. This study is an important step in the development of more effective treatments. Importantly, the study is the first to investigating binge eating disorder using a dimensional approach, by looking at the different aspects of the disorder, such as behavioral factors, biological factors, brain circuits and chemistry.

**Trial registration:**

Clinical Trials NCT02180984. Registered in July 2014.

**Electronic supplementary material:**

The online version of this article (doi:10.1186/s12888-015-0569-8) contains supplementary material, which is available to authorized users.

## Background

### Introduction

Binge Eating Disorder (BED) is characterized by recurrent episodes of excessive food consumption with experienced loss of control over eating and marked distress, which are not regularly followed by inappropriate compensatory behaviors [1]. Originally, BED was introduced in the 4th edition of the Diagnostic and Statistical Manual of Mental Disorder (DSM-IV) as a subcategory of Eating Disorder not Otherwise Specified (EDNOS) [2], but is now a separate diagnostic entity. The DSM-5 diagnostic criterion for BED includes significant changes. The minimum average frequency of binge eating (BE) has been reduced from at least twice/week for six months to at least once/week for three months.

BED is a clinically significant disorder affecting a broader range of individuals than anorexia nervosa (AN) and bulimia nervosa (BN) [3]. The lifetime prevalence of BED is higher in overweight and obese population [4], with numbers as high as 51 % of individuals seeking help in weight loss programs in Latin America [5]. In addition, BED is a long-term disorder, with a mean lifetime duration of 14.4 years [6] and, lastly, it is associated with significant impairment in quality of life, i.e. (both physical and mental health) [4, 7].

Despite the emotional and physiological consequences of BED, effective treatment associated with a long-term impact on weight is limited. Cognitive Behavioral Therapy (CBT) is currently the treatment of choice: it is reported to result in a 60 % long-term (one year) reduction in BE [8, 9]. However, with respect to weight loss, the impact of psychological interventions for BED is limited [10]. Similarly, despite reducing the frequency of BE, drug treatments are associated with little effect on weight, (with few exceptions such as sibutramine and topiramate [10–12]). Unfortunately, these medications are associated with safety restrictions: sibutramine has been withdrawn from the market in many countries, and topiramate has been associated with low tolerability [13]. Importantly, the paucity of information on the pathophysiological mechanisms underlying BED, is limiting further advances in treatment.

It is clear that, there is a need for a broader investigation of the mechanisms that are involved in the genesis and maintenance of BED, e.g. of the neurobiological processes underpinning bulimic behaviors and weight regulation. This type of exploration is in accordance with the US National Institute of Mental Health (NIMH) and the Research Domain Criteria (RDoC), which is a new research approach scientists are being encouraged to adopt it. In essence, the RDoC is a dimensional research approach that investigates mental disorders by looking at different aspects, such as behavioral factors, biological factors, brain circuits and chemistry [14]. The current project was based on the RDoC approach and guidelines; hence, different units of analysis and aspects of BED will be investigated.

### Psychoneuroendocrinological and immunological aspects of BED and obesity: food craving, stress, hormones and their association with control inhibition and compulsive eating

There is increasing interest in the conceptualization of disordered eating as a food addiction. This is substantially based on the fact that that addictions and bulimic type ED share phenomenological characteristics such as escalating frequency of the behavior, increased salience of food/drug stimuli, ambivalence towards treatment and frequent relapse [15, 16]. In terms of brain mechanisms, individuals who are drug addicts and obese (with and without BED), are reported to have decreased DA D2 receptor density in the striatal regions [17–19]: this may reduce the sensitivity to natural rewards and enhance impulsivity [20]. It has been hypothesized that impulsivity results from failures of response inhibition or ‘top down’ cognitive control involved in the activity of the prefrontal cortex (PFC), specifically the dorsolateral prefrontal cortex (DLPFC). Individuals with effective dietary self-control have increased activity in the left DLPFC when making decisions about food ingestions [21], suggesting that the DLPFC may be important for regulatory control over food consumption.

Abnormalities in the food reward system and BE may be associated with emotional and physiological stress. Cortisol, the primary effector of the hypothalamic-pituitary-adrenal (HPA) axis, may mediate stress-induced eating and play a role in disordered eating [22]. Stress is not only associated with disruption of the HPA axis, but may also sensitize pro-inflammatory responses known to take part in the pathogenesis of several ill-health conditions, including psychiatric disorders and obesity. For instance, the adipose tissue is part of the endocrine and innate immune systems [23]. During the fat storage process, adipose tissue produces inflammatory cytokines, such as C-reactive protein, TNF-alpha and IL-6. Importantly, adiponectin synthesis is reduced in obese individuals [24], and the mechanism that trigger the peripheral inflammation has a cascade effect in the hypothalamus [25], leading to the dysregulation in the physiological responses that maintains the sensitivity to insulin, leptin, ghrelin and peptide tyrosine tyrosine (PYY). Furthermore, stress and inflammation have been implicated in the disruption of synaptic signaling and integrity, a process mediated in part through the inhibition of neurotrophins’ function, in which BDNF is the most extensively characterized [26]. Beyond its importance in neural development and synaptic plasticity, there is evidence [27] indicating that BDNF is also essential for weight control and is involved in both homeostatic and non-homeostatic eating. In animal models [28], BDNF has anorexigenic effects in the hypothalamus.

Ovarian hormones are also involved in regulating food intake. Exacerbation of BE seems to occur when estrogen levels are low (premenstrual phase), and when progesterone levels are high (mid-luteal phase) [29]. Estrogen is mostly produced in the ovaries, but also in the liver, adrenal glands and fat tissue. Together with progesterone, ghrelin, leptin, PYY and BDNF, estrogen has a role in appetite regulation [30]. It is known that hormonal dysregulation, including estrogen, has an impact on mood, body weight and shape satisfaction, and on anxiety symptoms [31]. Further, estrogen has both anti-inflammatory and pro-inflammatory roles in the nervous system [32].

### Cognition & neuropsychological evaluation of BED

Neuropsychological research involving individuals with BED has demonstrated that in this group, cognitive performances are poorer on some executive functional tasks, such as, set shifting, inhibitory control [33], planning, decision making [34, 35], problem solving, attention, and working memory [36] than in healthy controls. However, the exactly cognitive profile and mechanism abnormalities of individuals with BED have not been established yet. Notably, the literature suggests that a similar cognitive impairment profile is present in obese individuals with or without BED, indicating that cognitive deficits may be related to obesity itself rather than to the ED [33, 34, 37], pointing out the need for further research.

Moreover, the use of neuropsychological instruments along with neuroimaging is important to identify mild cognitive impairments and create the opportunity to distinguish brain patterns associated with a specific disorder. A recent study [38] that used fMRI to investigate the neural correlates of inhibitory control in obese participants with BED (*n* = 35) found hypoactivity in brain regions involved in self-regulation and impulse control in obese BED participants during a cognitive control task (Stroop test) compared with obese non-BED and lean controls. The obese group with BED showed diminished activity in the ventromedial prefrontal cortex (vmPFC), inferior frontal gyrus (IFG) and insula. The authors concluded that the abnormalities in the activation of brain areas related to inhibition control in obese people with BED support the hypothesis that obese binge eaters differ from other obese individuals in regards to neurobiological aspects, including inhibitory control abilities. The study was the first to investigate the neural correlates of control inhibition in obese individuals with BED in comparison to lean controls.

The CPT-IP is a tool used to evaluate behavior inhibition and cognitive impulsivity [39]. A recent and small fMRI study with healthy subjects, using CPT to investigate brain regions involved during the CPT performance: it identified the left cerebellum and the frontal, temporal and occipital cortical brain areas. The ACC was identified with the greatest activation cluster during the same task [40]. Studies report that rTMS may lead to a better cognitive performance [41, 42], however further studies are necessary to understand its mechanisms of actions.

### Repetitive Transcranial Magnetic Stimulation (rTMS) for the treatment of BED

It has not been established whether the clinical benefit of any of the rTMS protocols in psychiatric disorders are a direct or indirect consequence of the modulation of neuronal excitability. It has been proposed that the associated release of neuromodulators and growth factors, e.g., brain derived neurotrophic factor (BDNF) [43], play a role in the mechanisms of action of rTMS [44].

There are pilot research studies on the effects of TMS on food cravings and bulimic behaviors in humans. A groundbreaking research study [45], demonstrated that rTMS applied to the left PFC might inhibit food craving compared to sham rTMS in healthy women considered “strong cravers.” An earlier small (*n* = 14) and unpowered, randomized, sham controlled trial found no benefit for the use of rTMS to control bingeing or purging behaviors in bulimic subjects [46]. In contrast, a subsequent proof-of-concept trial [47] reported positive findings of a one-off session of real rTMS compared to sham treatment for the reduction of food craving and BE in the 24 h follow-up in individuals with bulimic-type disorders. Furthermore, two recent reports have demonstrated rTMS to be an effective treatment for refractory bulimic-type ED. The first case report described a female with a diagnosis of both refractory BN and comorbid depression [48], and demonstrated that 20 sessions of rTMS (five sessions per week over four weeks) ameliorated depression symptoms, BE behaviors and purging, during the treatment. The second and most recent case report [49], described a young female diagnosed with refractory BED and comorbid depression. She was treated with 20 sessions of rTMS to the left DLPFC for 30 min/session at 10Hz for one month [2400 stimuli per day]),: this resulted in partial, but significant remission of depression symptoms and BE. Durability of clinical benefit of rTMS treatment has not been established, but studies that examined the short-term durability of rTMS effects on depressive symptomology demonstrated positive results [50, 51]. Repetitive TMS also holds promise as a tool for managing brain network plasticity properties and modulating cognitive abilities [52], and there is evidence that the intervention might be associated with up regulation of BDNF and possible neuroprotective effects [53].

Studies have also addressed the impact of rTMS on HPA axis function in humans [54, 55]. To date, the most consistent results are found for individuals with depression disorder. In this population, rTMS seems to reduce HPA axis activity. The effects of rTMS on the stress response in patients with bulimic-type ED have also been investigated in a double-blinded randomized sham-controlled trial study [47]: twenty-two female participants received a one-off session of high-frequency rTMS delivered to the left DLPFC, and salivary cortisol levels were assessed at four time points throughout the 90-min trial. Results showed that salivary cortisol concentrations of individuals receiving real rTMS were significantly lower compared to the individuals receiving sham rTMS.

Based on previous findings, rTMS is an important research tool to investigate the neurobiology of craving and BE. Thus, the current randomized controlled trial was developed to investigate the effects of rTMS on food craving and BE behavior, but secondary aims include the study of its effects on ED-related psychopathology (including depression, anxiety and stress symptoms), anthropometric measures, cognition, brain structure and function, hormones and inflammatory biomarkers. In addition, tolerability and safety of rTMS will be examined.

The study will provide the opportunity to enhance the knowledge on etiological similarities between addictions and bulimic disorders. It will also enable the investigation of the interplay of neurobiological mechanisms that are potentially involved in the pathogenesis of BED, and which might be vulnerable to the effects of rTMS.

### Hypothesis

Primary hypothesis:

The rTMS intervention will lead to the reduction of BE frequency and food craving.

The secondary hypotheses

- rTMS will promote a reduction in ED psychopathology (BES, TEF-Q, LOCES, BSQ,) and associated psychopathology (DASS-21, UPPS), a reduction of anthropometric measures (BMI, hip-waist circumference ratio) and an improvement in neurocognitive performance (Stroop test, executive functioning battery, particularly the CPT-IP).
- Following rTMS, plasma leptin and estrogen will be decreased along with cortisol levels and proinflammatory biomarkers (PCR, TNF-alpha, IL-6, IL-10) and that there will be an increase in plasma levels of ghrelin (in participants with >20 % of weight reduction), in levels of PYY and BNDF, as well as other anti-inflammatory biomarkers (adiponectin and IL-2).
- Participants will show that better inhibitory responses and greater activation in response inhibition regions (e.g. the DLPFC).
- There will be no differences between groups in prevalence of adverse events and dropout rates.
- Effects will be maintained at 8-weeks follow-up.

## Methods

### Overview and study design

**Fig. 1:**
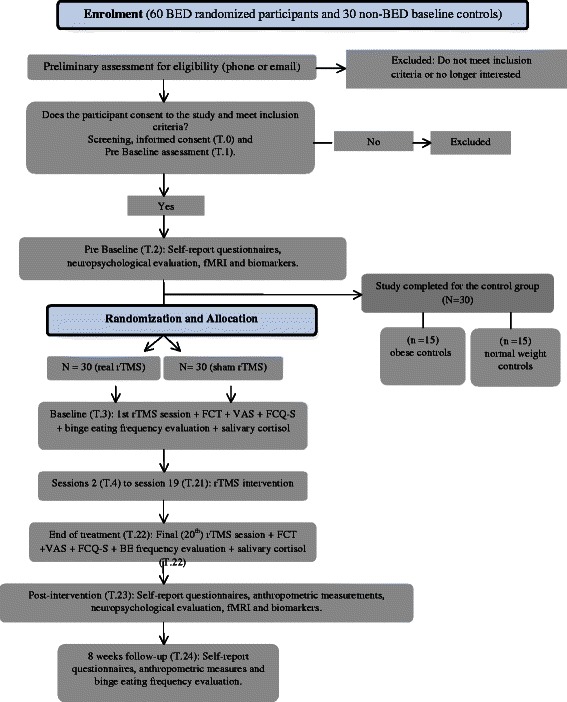
A summary of the study design and participants flow during the study

**Fig. 2:**
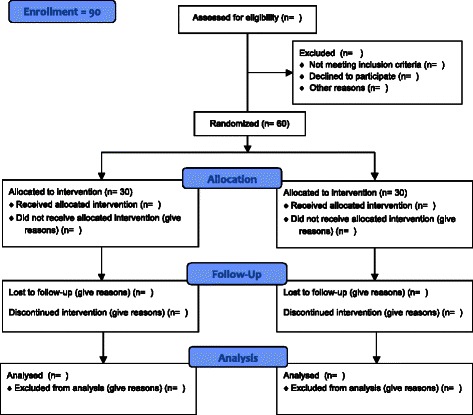
Consort 2010 Flow Diagram

This is a two-arm, double-blinded, randomized controlled trial (RCT). Sixty obese patients diagnosed with BED will be assigned to either: 1) a 20-session treatment protocol of real rTMS, or 2) a 20-session treatment protocol of sham rTMS. Participants will be evaluated for outcome measures before and after the intervention, with a follow up visit 8 weeks after the end of treatment. In addition, a control group comprising 30 female individuals with no lifetime diagnosis of ED (15 obese individuals without a diagnosis of BED and 15 normal weight healthy individuals) will be recruited and enrolled for baseline comparison (biomarkers, neuropsychological and neuroimaging evaluation). The trial design and study flow are shown graphically in Figs. 1 and 2, in line with the SPIRIT 2013 Statement [56].

### Participants

The study will be conducted at a specialized outpatient program for eating disorders (ED) of the Federal University of São Paulo (UNIFESP), Brazil. Participants will be females seeking treatment for their ED or referred by their clinical care providers. Recruitment will also include individuals attending online advertisement, study flyers, and newsletters.

All participants will be informed about the study and asked to sign the informed consent term before any procedure is conducted. Study intervention will be completed in approximately 7 weeks.

### Enrollment

#### Inclusion criteria for randomization

Participants are eligible for randomization if they fulfill the following inclusion criteria:

1. Meet DSM-5 criteria for BED.
2. Age between 18 to 55 years old.
3. Right-handed and able to write, read, and understand all elements of the study.
4. Females.
5. BMI ≥ 35 kg/m^2^ and body weight ≤ 150 kg.
6. Laboratory blood tests (fasting glucose, fasting glucose/insulin ratio, complete blood count, TSH and T4) within normal range at study enrollment. In order to exclude participants with diabetes, fasting glucose levels under 126 mg/dl will be accepted.

#### Inclusion criteria for controls

Control participants are eligible if:

1. BMI between 19 and 24.99 kg/m^2^ for normal weight controls and BMI ≥ 35 kg/m^2^ (and less than 150 kg) for obese controls.
2. Normal menstrual cycle (every 21–35 days) for the past 6 months.
3. Age between 18 to 55 years old.
4. Right-handed and able to write, read, and understand all elements of the study.
5. Females.
6. Laboratory blood tests (fasting glucose, fasting glucose/insulin ratio, complete blood count, TSH and T4) within normal range at study enrollment.

#### Exclusion criteria

1. History of head or eye injury or epilepsy.
2. Body metallic implants, pacemaker, claustrophobia and any other contraindication to fMRI or rTMS.
3. Current use of psychotropic drugs (except for antidepressants on a stable dose for at least one month).
4. Current use of any anti-obesity drug (e.g. sibutramine, orlistat) and medications that are known to reduce weight, such as Liraglutide and Topiramate (three months washout period for any medication).
5. Pregnant or breastfeeding.
6. Diabetes Mellitus diagnosis.
7. Major psychiatric disorder requiring immediate treatment (e.g. Schizophrenia, Bipolar Disorder).
8. Substance dependence (smokers of less than 5 cigarettes/day will be included).
9. Individuals currently receiving any psychological therapy for ED.
10. Cushing’s and Turner’s syndrome.

*Control participants will be excluded if they meet psychiatric disorder criteria based on the MINI International Neuropsychiatric Interview 7.0 [57].

#### Withdrawal criteria

Participants will be withdrawn from the study if they: i) become pregnant; ii) experience any adverse event which the study investigator specifies as an indicator that it is no longer safe for the individual to participate; iii) initiate the use of any medication described as an exclusion criteria; iv) develop psychotic or manic symptoms, or v) failure to complete three consecutive rTMS sessions.

### Intervention

Following assessment, participants eligible for the study will undergo a preliminary fMRI, followed by 20 sessions of neuronavigated rTMS, one session per day, 3 days/week over approximately 7 weeks. Focal rTMS will be performed using a Neurosoft device and a ‘figure of eight’ coil. Brainscience Neuronavigation will be used to guide the placement of the coil to the target PFC region using a template MRI for all participants. The coil will be placed at a 45° angle to the mid-sagittal line to induce a posterior to anterior current in the underlying neural tissue. For the real treatment condition, stimulation will target the left DLPFC at 110 % of the resting motor threshold. Each session of 10 Hz stimulation will apply 1000 pulses to the left hemisphere, with a duty cycle of 5 s on and 55 s off, for a total stimulation time of 20 min. Sham rTMS treatment will be administered with the same TMS methodology used for real intervention, except for the fact that in the sham condition there is no actual magnetic stimulation. In order to mimic the cutaneous sensation and muscle twitching of rTMS without stimulating the brain, focal electrical stimulation will be used as a sham rTMS condition. To ensure treatment adherence, if rTMS sessions are missed, replacement sessions will be schedule within a week.

### Measures

Additional file 1: Table S1 describes the study measures and timeframe in details.

#### Diagnosis and eligibility measures

##### Anthropometric measures

Height and weight will be measured in triplicate using a stadiometer and a scale. Participants will be backwards and in a hospital gown. Hip-waist circumference and ratio will be measured in triplicate also.

##### Laboratory blood examination

Fasting blood samples will be obtained for a complete blood count, fasting glucose levels, fasting glucose/insulin ratio, TSH, and free thyroxine (T4). A urine pregnancy test will be collected if pregnant condition is possible (women in fertile age and not using a reliable contraceptive method).

##### Diagnostic interviews

Psychiatric diagnosis will be assessed with The Structured Clinical Interview (MINI 7.0). The interview comprises modules representing different groups of psychiatric disorders. In order to confirm BED diagnosis (DSM-5) and collect further information on ED psychopathology, The Eating Disorder Examination (EDE 17.0D) will also be applied. The EDE is a semi-structured diagnostic interview and the gold-standard instrument used in the diagnosis of eating disorders [58]. It has four subscales of symptoms severity (shape, weight and eating concern and dietary restrain) and a global score. In addition the number of binge eating episodes and days during the past month (or 28 days) is carefully assessed during the interview. Trained professionals will be conducting these interviews.

##### TMS/fMRI safety questionnaires

These questionnaires will be administered in order to rule out potential contraindications to TMS intervention and/or fMRI exams, such as, history of epilepsy, metal implanted devices, brain or eye injury, pregnancy, stroke, etc.).

##### Clinical measures

Medication use and menstrual status will be assessed based on a questionnaire developed by the authors.

#### Primary outcome measures

The primary outcomes of this study are: (1) the change in the number of BE episodes before and after study treatment (number of BE episodes at baseline subtracted from the number of BE episodes at the end of treatment), as measured by participants recording of binge episodes in the food diary during the previous 15 days to the baseline visit (first rTMS session, T.3) to the end of treatment visit (T.23); (2) the change in “urge to eat” (craving) as measured in a 10 cm VAS (from T3 to T22).

#### Secondary outcome measures

Self report measures

1. A short and modified version (comprising 8 items) of the Food Craving Questionnaire-Trait (FCQ-T) [59]. The FCQ-T was translated and adapted to Brazilian Portuguese (publication in progress). This instrument investigates various aspects of food cravings across different time periods and situations.
2. Binge Eating Scale (BES) [60]. BE severity will be examined using the BES, which is composed of 16 items and has been translated and validated to Portuguese Change in the total mean score from the baseline visit (T.3) to the end-of treatment evaluation (T.23) and 8-week follow up (T.24) will be compared between the two randomized groups.
3. Three Eating Factor Questionnaire (TEF-Q) [61]. The reduced 21 item version (TEFQ-R21) [62], which has been translated and adapted to Brazilian Portuguese [63] will be used. Overall, the questionnaire evaluates the following aspects: eating cognitive restrain, emotional eating and control over eating.
4. The Loss of Control over Eating Scale (LOCES) [64] is an instrument developed to investigate aspects associated with the loss of control over eating. The scale has been translated and validated to Brazilian Portuguese (publication in progress).
5. UPPS Impulsive Behavior Scale [65] was originally developed with five factors and later reduced to four factors and 45-items [66]. These factors were divided as: a) urgency, tendency to experience strong impulses, b) lack of premeditation, tendency not to think about the consequences of an act before engaging in it, c) lack of perseverance, lack of the ability to stay focused on a task that can be boring or difficult, and d) sensation seeking, tendency to search for activities that are exciting, and openness to try new experiences. The scale is also available in Brazilian Portuguese [67].
6. Depression Anxiety Stress Scales (DASS-21) is a 21-item self-report scale [68] developed to measure the negative emotional states of depression, anxiety and stress during the past week. Each item is rated using a 4-point severity/frequency scale according to the corresponding statement. DASS-21 is available in Brazilian Portuguese [69].
7. Food diary. Participants will be instructed to register complete food intake in a food diary for seven consecutive days before the T.3, T.23 and T.24 visits, and to record BE episodes for 15 consecutive days before the same time points (T.3, T.23 and T.24).
8. Body Shape Questionnaire (BSQ-34). The questionnaire has been validated in patients with eating disorders and includes assessments of body image. The questions related to body shape perception and range from 1 to 6. Higher scores on these scales indicate greater psychopathology. The questionnaire has been translated and validated to Brazilian Portuguese [70].
9. The 12-item Short Form Health Survey (SF-12). This questionnaire measures health-related quality of life, subdivided in two scales – Physical Health Component Summary scales (PCS) and Mental Health Component Summary scales (MCS) [71]. It has been translated into Brazilian Portuguese [72].

Anthropometric measures will be completed as previously described.

##### Biomarkers

Blood serum levels of reactive-C protein, BDNF, adiponectin, TNF-Alpha, IL-6, IL-10, leptin, ghrelin, PYY_3–36_, estradiol and progesterone will be measured in order to analyze changes in inflammatory response, hormones and neurotrophic factors.

##### Cognitive evaluation

The evaluation will include seven cognitive tests investigating executive functions such as, working memory, control inhibition and decision-making, as described in details in Additional file 2: Table S2.

##### Neuroimaging

Neuroimaging scans will be performed on a 3 T Magneton Trio Tim System (Siemens, Germany) scanner. Magnetic Resonance Imaging (MRI) scans will be acquired for the whole brain structure (T1-weighted, 1 mm resolution). Functional Magnetic Resonance Imaging (fMRI) scans for resting state (no task) will be acquired to investigate functional connectivity. T2-weighted echo-planar images (EPI) depicting blood oxygen level dependent (BOLD) contrast will be acquired during the Stroop Word Color Task (SWCT). The SWCT is widely used as an index of attention and executive control [73]. The task requires the ability to actively inhibit a learned response in favor of a more voluntary (and difficult) response. A SWCT block-design of three conditions (control/congruent, neutral and incongruent) will be used during the fMRI. Neuroimaging allows for a deeper understanding of brain structures, patterns and activation. The fMRI exam and the SWCT will be used as tools to investigate brain areas involved in automatic inhibitory responses during the SWCT. Further, the SWCT allows the investigation of differences in activation patterns and connectivity in the PFC during the task [74].

Food Challenge Task (FCT): The task was used and described in previous studies [47]. The FCT requires participants to watch a film clip (2 min.) followed by exposure of food (10 min.), in order to stimulate craving. The current study will only use the film clip to stimulate craving; there will be no food exposure. Participants will also complete the Visual Analogue Scale (VAS) of “urge to eat”, “hunger”, “tension” and “mood” and a short and modified version (comprising 5 items) of the Food Craving Questionnaire-State (FCQ-S) [59], which was translated to Brazilian Portuguese and is in the process of validation. The FCQ-S investigates aspects of food craving in real time. In addition, salivary cortisol samples will be collected along with the FCT to examine potential impact of stress (exposure to food cues).

##### rTMS tolerability and safety

Participants will be provided with a monitoring record and instructed to record any experience of adverse events during the rTMS treatment. The monitoring record will be reviewed before each rTMS session. Tolerability will be based on the number of adverse events reported (grouped per side effect type) and number of dropouts due to adverse events or other reasons. Safety will be measured based on the occurrence of serious adverse events, such as seizures (reported as rare in rTMS studies).

### Sample size and statistical analysis

To detect the change in craving and BE frequency before and after treatment intervention, the following parameters were used to estimate sample size: effect size *f* = 0.31 (supposed to be small), alpha error probability =0.05, power = (1-β error probability) = 0.8, number of groups = 2, number of measurements through the follow-up = 3 and correlation among repeated measurements = 0.5.

Statistical Inferences: Two different types of analyses will be used to evaluate the effectiveness of the rTMS. The first (and standard) method is intention-to-treat (ITT) that assumes that every patient allocated to be intervention actually received it [75]. The other paradigm, CACE estimation method [76, 77] will take into account the compliance status (patient’s adherence to the rTMS).The compliance status is defined here as at least one of 20 session of rTMS; CACE estimation, therefore, provides a realistic effect under non-adherence phenomenon being estimated based on the structural equation modeling. Underlying both paradigms, it will be used growth modeling, which examines the development of individuals on one more outcome variables over the time. In growth modeling (on contrary of repeated ANOVAS), random effects are used to capture individual differences in development. The corrected significance level (Bonferroni) to be adopted due to 32 outcomes will be 0,0015. Such procedure will be carried to avoid false discovered rate due to multiple comparisons. All the statistical inference will be done via Mplus 7.0. In addition, careful fMRI image acquisition and processing will be completed and analyzed taking into consideration the limitations of fMRI studies.

### Randomization

A simple randomization will be conducted with a 1:1 allocation. A randomization sequence list will be created using the website www.random.org, which offers true random numbers; such randomness comes from atmospheric noise, which for many purposes is better than the pseudo-random number algorithms typically used in computer program. Participants will be randomly assigned following simple randomization sequence to one of the two treatments. Group 1 for active rTMS and group 2 for sham rTMS. Patient allocation will be blinded to all participants and study staff, in exception to the professionals providing the rTMS treatment and one doctorate student, responsible for allocation concealment. The allocation sequence will be concealed and sequentially numbered in dark, sealed and stapled envelopes and locked in a key protected cabinet file. Allocation implementation will occur pre-intervention and between the T.2 and T.3 visits.

### Ethical review and trial registration

The protocol has been reviewed and approved by Research Ethics Committee UNIFESP/EPM in São Paulo, Brazil (registration number: 26164614.7.0000.5505). It is registered in www.clinicaltrials.gov (trial ID: NCT02180984).

## Discussion

A major criticism of descriptive approaches to psychiatric nosology concerns the lack of agreement between current diagnostic categories and findings from neuroscience research. The current descriptive diagnostic system might not comprise true phenotypes, as most genetic findings and neural circuit maps appear either to link to several different currently described psychiatric disorders or to different subgroups within syndromes. In response, efforts to incorporate neurobiological-salient dimensions into the classification of psychiatric disorders have received increasing emphasis in recent years. The Research Domain Criteria (RDoC) initiative is an example of this new perspective. The RDoC was conceived as a dimensional research approach, which views mental disorders as brain disorders [14], and investigates mental disorders by looking at different aspects, such as behavioral and biological factors, brain circuits and chemistry.

The current proposal is to use rTMS as a research tool to advance in the understanding of the different aspects altered in BED within the RDoC framework. Thus, the aim of this study is to extend the investigation of the effects of rTMS on craving and ED psychopathology to the exam of patterns of comorbidity, neurobiological and neuropsychological underpinnings, and of potential biomarkers. Functional approaches, such as the evaluation of resting state connectivity and task-induced activation, have great potential to identify targeted neurocognitive diagnostic markers and may indicate illness severity and prognosis with increased accuracy. This is the first randomized clinical trial to investigate the rTMS treatment effects in obese women with BED. Previous studies had limitations such as small sample sizes, absence of follow up data and lack of a dimensional approach. In response to the current shortcomings in knowledge of BED etiology and the limited treatments available, we hope to support innovation in the development of more targeted and effective evidence-based treatments.

## Additional files

Additional file 1: Table S1. A summary of instruments and measures completed at each study time point. (PDF 75 kb)
Additional file 2: Table S2. Cognitive evaluation: A short description of cognitive tests used for evaluation. (PDF 52 kb)

## Abbreviations

AN: Anorexia Nervosa
ANOVAS: Analysis of variances
BE: Binge eating
BED: Binge eating disorder
BDNF: Brain derived neurotrophic factor
BN: Bulimia Nervosa
BES: Binge eating scale
BMI: Body mass index
CACE: Complier average causal effect
CBT: Cognitive Behavior Therapy
DLPFC: Dorsolateral pre-frontal cortex
DSM: Diagnostic Statistical Manual
ED: Eating Disorder
EDNOS: Eating disorder not otherwise specified
FCT: Food challenge Task
FCQ-S: Food craving questionnaire state
FCQ-T: Food craving questionnaire trait
fMRI: Functional Magnetic Resonance Image
HPA: Hypothalamic-pituitary-adrenal
Hz: Hertz
IFG: Inferior frontal gyrus
ITT: Intent to treat
LOCES: Loss of control over eating scale
PFC: Pre frontal cortex
PYY: Peptide tyrosine tyrosine
rTMS: repetitive transcranial magnetic stimulation
RCT: Randomized clinical trial
RDoC: Research Domain Criteria
TMS: Transcranial magnetic stimulation
VAS: Visual analogue scale
VMPFC: Ventromedial prefrontal cortex.
