# Supplementary material for: The effects of repetitive transcranial magnetic stimulation in obese females with binge eating disorder: a protocol for a double-blinded, randomized, sham-controlled trial
Source: BMC Psychiatry. 2015 Aug 12;15:194. doi: 10.1186/s12888-015-0569-8 (PMC4533762; doi:10.1186/s12888-015-0569-8)
Supplement: Additional file 1: Table S1. — A summary of instruments and measures completed at each study time point. (PDF 75 kb) [file 12888_2015_569_MOESM1_ESM.pdf]

Table 1: Study measures and time points

Measure	Screen (T.0)	Pre Baseline 1 (T.1)	Pre Baseline 2 (T.2)	Baseline rTMS Session 1 (T.3)	rTMS Sessions 2 to 19 (T.4-T.21)	rTMS Last Session 20 (T.22)	End-of Treatment Evaluation (T.23) – within 1 week after the last rTMS session.	8 week follow up (T.24)
Informed Consent	✓							
Height & Weight<br>Hip-waist circumference	✓			✓			✓	✓
Menstrual history	✓						✓	
Evaluation of the Presence of Binge Eating & MINI (diagnostic interview)	✓							
Evaluation of Binge Episodes & Adverse Events *(at each visit – occurrence between visits)*				✓	✓	✓		✓
EDE 17.OD		✓						
Laboratory blood exams		✓						
Biomarkers & fMRI			✓				✓	
TMS safety questionnaire	✓							
7-day food intake diary (Completed 1-week before T.3, T.23 and T.24 visits)				✓			✓	✓
Binge eating recording (in diary, 15 days before T3, T23 and T24				✓			✓	✓
Self-report Questionnaires* (completed 1 week before the visits T3, T23 and T24)				✓			✓	✓
Cognitive evaluation			✓				✓	
FCT + VAS + FCQ-S and salivary cortisol				✓		✓		

EDE = Eating Disorder Examination; FCT = Food Challenge Task; fMRI = Functional Magnetic Resonance Image; rTMS = Repetitive Transcranial Magnetic Stimulation; *Self-report questionnaires: Binge Eating Scale (BES), Depression Anxiety Stress Scales (DASS-21), Food Craving Questionnaire-Trait (FCQ-T), The Loss of Control over Eating Scale (LOCES), Three Eating Factor Questionnaire (TEF-Q), UPPS Impulsive Behavior Scale; T. = Time; VAS = Visual Analogue Scale.
