# Supplementary material for: The effects of repetitive transcranial magnetic stimulation in obese females with binge eating disorder: a protocol for a double-blinded, randomized, sham-controlled trial
Source: BMC Psychiatry. 2015 Aug 12;15:194. doi: 10.1186/s12888-015-0569-8 (PMC4533762; doi:10.1186/s12888-015-0569-8)
Supplement: Additional file 2: Table S2. — Cognitive evaluation: A short description of cognitive tests used for evaluation. (PDF 52 kb) [file 12888_2015_569_MOESM2_ESM.pdf]

Additional file 2: Neuropsychological evaluation

Instruments	Cognitive skills	Descriptions	Duration
Iowa gambling task (IGT) (CV)	Decision-making.	Card game involving choosing among many different piles of cards in order to win money, however sometimes there is the loss of money.	15 minutes
Continuing performance test (CPT) (CV)	Sustained and selective attention.	The test involves monitoring a sequence of numbers appearing on the computer screen. Participants react (pressing a bottom) every time identical numbers appears in the sequence.	10 minutes
Number letter task (CV)	Cognitive flexibility.	Pair of cards (letters and numbers) is presented and should be classified as odd or even, vowels or consonants according to the instruction.	5 minutes
Keep track task (CV)	Working memory.	Some words are classified into different categories (family, animals, color, countries, distance, metals). Different lists of these words are presented six times. The participant is required to remember the last word of each category.	10 minutes
Vocabulary (WAIS-III)	Estimated IQ intelligence test/vocabulary.	Sets of words are read to the participant by the examiner and the participant is required to give the word definition.	10 minutes
Matrix reasoning (WAIS-III)	Estimated IQ intelligence test/perceptual learning.	Participant is presented with an incomplete matrix and is required to complete the matrix by choosing one of the five options available.	10 minutes

CV: Computerized version.
